# Testing the Neutral Theory of Biodiversity with Human Microbiome Datasets

**DOI:** 10.1038/srep31448

**Published:** 2016-08-16

**Authors:** Lianwei Li, Zhanshan (Sam) Ma

**Affiliations:** 1 Computational Biology and Medical Ecology Lab, State Key Lab of Genetic Resources and Evolution, Kunming Institute of Zoology, Chinese Academy of Sciences, Kunming, China

## Abstract

The human microbiome project (HMP) has made it possible to test important ecological theories for arguably the most important ecosystem to human health—the human microbiome. Existing limited number of studies have reported conflicting evidence in the case of the neutral theory; the present study aims to comprehensively test the neutral theory with extensive HMP datasets covering all five major body sites inhabited by the human microbiome. Utilizing 7437 datasets of bacterial community samples, we discovered that only 49 communities (less than 1%) satisfied the neutral theory, and concluded that human microbial communities are not neutral in general. The 49 positive cases, although only a tiny minority, do demonstrate the existence of neutral processes. We realize that the traditional doctrine of microbial biogeography “*Everything is everywhere, but the environment selects*” first proposed by Baas-Becking resolves the apparent contradiction. The first part of Baas-Becking doctrine states that microbes are not dispersal-limited and therefore are neutral prone, and the second part reiterates that the freely dispersed microbes must endure selection by the environment. Therefore, in most cases, it is the host environment that ultimately shapes the community assembly and tip the human microbiome to niche regime.

Hubbell’s^1^ neutral theory presented a null model for testing the mechanisms of species coexistence and biodiversity maintenance in ecological communities. It also offered a simple mechanistic explanation of the species abundance distributions (SAD), which has been extensively studied in community ecology since the 1940s, but still lacks a theoretical consensus for their interpretations^2^,^3^,^4^,^5^,^6^,^7^,^8^. Neutral theory contrasts with the traditional niche theory^9^,^10^,^11^,^12^,^13^,^14^ by assuming that the properties of individuals in a community are independent of their species identity. The traditional niche theory would assume that individuals of different species occupy different niches due to their species-specific properties and the niches are of limited similarities. According to neutral theory, local communities are built by random draws from regions pools driven by stochastic colonization, and the deterministic competitive interactions are insignificant in shaping the local community compositions because species are supposed to be competitively equivalent^1^,^15^,^16^. The neutral theory also assumes that abundant regional species possess a higher colonization probability and consequently the relative abundance of each species in a local community should mirror its abundance in the regional community at any given time^1^,^16^. Consequently, neutral processes driven community should be less influenced by the differences in environmental factors, and demonstrate less of a direct link between the environment and assembly processes^17^. The significance of Stephen Hubbell’s neutral theory of biodiversity^2^ is multifaceted, whether or not the null model fits to a specific community. For example, as demonstrated in this article, the traditional view that “*everything is everywhere*, *but the environment selects*” originally proposed by Baas-Becking^18^ is a well-known doctrine but receiving occasional challenges especially in recent years, indeed make great sense from the neutral theory perspective.

During the past decade, there has been extensive testing of the neutral theory, mostly with macro community ecology data^19^,^20^,^21^,^22^,^23^. McGill^21^ reviewed empirical tests of neutral theory; many of the tests used the neutral theory model as a null model and used another model (often lognormal distribution model) as the alternative model. However, in many cases, the alternative model was loosely defined, and hence the rejection of neutral theory could not automatically prove that a particular alternative niche-based model holds. In fact, the approach of using simplified assumptions, which are not required to be strictly accurate, about complex systems to improve understanding and to see what can be explained with the simplified models is a well-accepted practice in many fields of sciences^24^. Therefore, the primary value of the neutral theory is not whether or not it can adequately describe a particular dataset because the failure itself can inform us that processes other than the neutral processes prevail in shaping the structure of the community under investigation.

The neutral theory was developed and tested primarily in the context of ecological communities of plants and animals. In contrast to the many macro-ecological studies, there are relatively few applications of neutral theory in microbial ecology^17^,^18^,^25^,^26^,^27^,^28^,^29^,^30^,^31^,^32^,^33^,^34^,^35^,^36^,^37^,^38^. The scarcity is primarily due to the reality that majority of microbes are not cultivable, and their detections were extremely difficult until the invention of the metagenomic technology nearly a decade ago, which revolutionized the techniques for studying microbial community ecology^39^,^40^,^41^,^42^,^43^. When majority of the microbes could not be counted, or even detected, it would be very difficult if not impossible to collect sufficient data for testing ecological theory, including the neutral theory. Today the wide availability of metagenomic technology and the on-going Human Microbiome Project (HMP) offer us an unprecedented opportunity to apply and test major ecological theories for the studies of microbial communities^43^.

Limited tests of the neutral theory with microbial communities have produced mixed results in the existing literature. Earlier studies in environmental microbiology were mostly positive, supporting the neutral theory (*e.g*. refs 26, 27, 28), but more recent studies, especially those studies on the human and animal microbiomes seem to be mixed with more negative cases^17^,^30^,^32^,^36^,^44^ than positive cases^35^,^37^. Sloan *et al*.^26^ and Woodcock^37^ were among the first who explored the roles of immigration and chance in shaping prokaryote community structure. Woodcock^28^ performed the first comprehensive testing of the neutral theory in microbial ecology, and presented strong evidence that neutral community models fitted to the microbial communities in tree holes filled by rain water^28^,^45^ conjectured that the scale of regional communities in microbes might be different from that of macro-communities since the dispersal distance of microbes can be more extensive. In practice it is most likely that both niche and neutral mechanisms are in effect in microbial communities^46^,^47^,^48^,^49^,^50^, but niche effect is often more prevalent than neutral effect^46^,^47^. Of course, there were also the opposite cases, *i.e.,* neutral effect is more significant (*e.g.* ref. 48).

The potential importance of neutral process in shaping the human microbiota has been conjectured in some perspective reviews^43^,^51^, but a handful of existing tests of the neutral theory with the human microbiome (including animal microbiome) only offer conflicting evidence. Jeraldo^47^ tested the role of neutral process in structuring the gut microbiome of three domesticated vertebrates, and they concluded that, although the species abundance patterns were seemingly well fit by the neutral theory, the theory couldn’t explain the evolutionary patterns in the genomic data (*i.e*., phylogenetic diversity). Furthermore, their analyses strongly supported the non-neutral (niche) role in shaping the animal microbiomes. Avershina *et al*.^31^, through a study of the faecal microbiota with 16S rRNA sequencing technology from a healthy cohort of 86 mothers and their children, observed a clear age-related colonization pattern shift in children and suggested that neutral processes are involved in shaping the gut microbiota. In contrast, there was not a similar shift in microbial composition during mother’s pregnancy. This finding is not consistent with previously mentioned finding from Jeraldo^47^ study on the gut microbiome of three domesticated vertebrates. Avershina *et al*.^31^ suggested that the inconsistence could be reconciled by the fact that niche selection should limit the phylotypes allowed in a given environment (the gut), whereas among the allowed phylotypes, neutral process could be in effect. Levy & Borenstein^32^ demonstrated the non-neutral assembly processes and complex co-occurrence patterns in the human gut microbiome. Guan & Ma^44^ tested the neutrality of human milk microbiome, and the test results were negative. Schmidt *et al*.^17^ found that deterministic processes appear to govern the assembly of fish microbiome, and stochastic colonization does not occur in their system. In the above four case studies^17^,^32^,^44^,^47^, the human or animal microbiome datasets failed to support the neutral theory.

Besides above mentioned gut microbiome study by Avershina *et al*.^31^, the following two case studies also presented supporting evidence to the neutral theory. Morris *et al*.^35^ applied the neutral theory to test whether dispersal from the mouth (source community) can adequately describe the observed microbial distribution in the lung. They also tried to identify microbial groups that significantly deviate from neutral community pattern, and they conjectured that these microbes might be advantageous in the lung habitat. They discovered that lung bacterial populations were similar to those in the oropharynx, but the lung does have some distinctive bacterial populations, not from contamination or dispersal from the mouth. Nevertheless, the functions of these lung habitat specific bacteria as well as the nature of the immune response to them are open for further research. Venkataraman *et al*.^37^ further utilized the neutral theory model to distinguish the microbes dispersing to the lungs from other body sites and atmosphere from the microbes indigenous to the lungs, assuming that the former (“neutrally distributed” species, *i.e*., consistent with dispersal and ecological drift) can be predicted with a neutral model and consequently the latter can be predicted from the difference between the actual presence and the former. They also suggest that the non-native microbial species should have a competitive advantage to the indigenous lung microbes. Their study reveals that the bacterial community composition of the healthy lung can be satisfactorily predicted with the neutral model, reflecting the overriding role of dispersal of microbes from the oral cavity in shaping the microbial community in healthy lungs. In contrast, it is not the case with the microbiome of diseased lungs where active selection is underway. Therefore, the bacterial neutral distribution is a distinguishing characteristic of the microbiome in healthy lungs. But, bacterial populations in diseased lungs appear to be under active selection. The ability to measure the relative importance of selection and neutral processes including dispersal in shaping and maintaining the healthy lung microbial community is a critical advance for understanding its influence on host health^37^. Ding & Schloss^52^ demonstrated that it is possible to distinguish different community types despite considerable intra-subject and inter-subject variation in the human microbiome, which has important implications for assessing disease risks associated with certain community types. If neutral theory can be utilized to distinguish healthy and diseased microbial communities, such as demonstrated by Venkataraman *et al*.^37^, its practical biomedical significance is self-evident.

More recently, advances have also been made in the theoretical exploration of the neutral process in microbial communities. Holmes *et al*.^30^ and Harris *et al*.^36^ reformulated Hubbell^2^ neutral theory model as a hierarchical Dirichlet multinomial mixture process to simulate the human gut microbiome. They concluded that functional niches mainly determine the human gut microbiome, and neutral assembly may only play a partial role in shaping the fine-scale gut microbial diversity—operating within the species occupying a specific function niche (*i.e*., those species playing a same metabolic role). They termed those cases “borderline” neutral patterns. They also discovered a negative correlation between body mass index (BMI) and immigration rates within the family Ruminococcaceae^36^, which offers a freshly new interpretation of the relationship between obesity and gut microbiome. Harris *et al*.^36^ not only revealed these important insights on gut microbiome but also developed a general computational procedure to efficiently fit multisite neutral theory models. We will further discuss this significant methodological advance in the discussion section.

O’Dwyer *et al*.^33^,^34^ tested the neutral theory from phylogenetic diversity perspective, rather than traditional species diversity perspective, with human microbiome data. Interestingly, they found a clear impact of metacommunity size (scale) on the phylogenetic diversity of body habitats relative to the null hypothesis. They also found that whole microbiome diversity for a given subject is typically much lower than a random sample from the metacommunity, which is complicated by a wide range of different behaviors for the distinct habitats within that subject. Overall, they concluded that there are significant variations in diversity across habitats relative to the prediction from the neutral theory. Zeng *et al*.^38^ developed an agent-based architecture that encapsulated the neutral model of community diversity with added dimensions of an evolutionary timescale and a genealogy of hosts. This is essentially a simulation system that can be utilized to test various assumptions on community assembly including neutral theory assumptions and its advantages include the consideration of the parental contribution effects on community assembly process as well as how the process operates over evolutionary time.

The objective of this study is two-fold: First, we conduct a comprehensive testing, with extensive HMP datasets covering all five major body sites (gut, oral, skin, vaginal, and nasal) of the human microbiome, including 7437 communities from 18 locations of 242 individuals, and with rigorous statistical simulation tests to answer the question—is the community assembly in the human microbiome neutral? Second, we discuss the implications of the neutral theory to understanding the human microbiome diversity and explore the underlying mechanisms if the data fail to support the neutral theory.

## Materials and Methods

### The 16s rRNA sequence datasets of human microbiome

The datasets we use to test the neutral theory were obtained from HMP data center (www.hmpdacc.org). Specifically, we selected the 16s rRNA sequence datasets of 18 sites of 5 locations sampled from 242 subjects^42^ (termed 240-healthy-subjects HMP datasets, hereafter). Those body sites and locations are: airways (anterior nares), gut (stool), oral (attached keratinized gingival, buccal mucosa, hard palate, Palatine tonsils, saliva, subgingival plaque, supragingival plaque, throat, tongue dorsum), skin (left antecubital fossa, left retroauricular crease, right antecubital fossa, right retroauricular crease), and urogenital (mid vagina, posterior fornix, vaginal introitus). We used the datasets of both V1-V3 region and V3-V5 region in our analyses, and totally 7437 community samples (V1-V3, 2855 samples; V3-V5, 4582 samples, respectively) were sequenced, and two collections of OTU tables (corresponding to V1-V3, and V3-V5 regions, respectively) were obtained from the 16s-rRNA mothur software pipeline. Each sample corresponds to one row in the OTU tables, and is used to fit the neutral theory model. The OTU tables were computed with a cutoff 3% dissimilarity based on RDP database and they are available for download at www.hmpdacc.org. Those OTU tables contain species abundances (OTU reads) within each community sample.

### The computational procedures

The essential aspects of the neutral theory can be summarized as: (*i*) it assumes that interacting species are equivalent on an individual ‘per capita’ basis, (*ii*) it is an individual-based stochastic dynamic theory, (*iii*) it is a sampling theory, and finally (*iv*) it is a dispersal-assembly theory^53^. The theory reveals the significance of dispersal limitation, speciation and ecological drift in community assembly and maintenance. As a null model, it offers us an apparatus to evaluate the role of adaptation and natural selection in the context of evolutionary community ecology^54^. The classic neutral theory model describes a local community containing *J* individuals. One of these individuals, chosen at random, dies and is replaced at every time step. The replacement can be either an offspring of another randomly chosen individual from the local community, occurring with probability *1-m*, or an offspring of a randomly chosen individual from the metacommunity, with probability *m*. The parameter *m* is considered as a measure of *dispersal limitation*. A problem with *m* is that it does not translate into dispersal limitation in the most logical way; for example, a small local community may involve a much smaller flux of immigrants for the same value of *m*. For this reason, an alternative parameter *I* is often used instead of *m*^55^.

We use two sampling formulae for testing the neutral theory: one was proposed by Ewens^56^, and another by Etienne^57^ that was also inspired by Ewens formula. Both are accurate likelihoods for different scenarios, and Etienne’s one is for the best-known model of Hubbell’s^2^ neutral theory. Etienne’ s formula adds dispersal limitation to Ewens formula, and we can infer that dispersal limitation plays a significant role in neutral community assembly if Etienne’s formula performs better than Ewens formula. In literature, the comparisons between Ewens and Etienne and sampling formulae showed that the latter outperformed the former when the dispersal limitation plays a significant role in community assembly^57^.

We will first compare the *log-likelihoods* computed with Ewens formula and Etienne formula to determine which of them is better suited for the human microbiome datasets. We then compare the *log-likelihoods* of the observed community (sample) and neutral-theoretic community to test whether or not the neutral theory model fits to the observed community, based on either Ewens^56^ or Etienne^57^, whichever performed better in the previously described first step. In addition, we also hope that the possible difference between Ewens^56^ or Etienne^57^ and log-likelihoods will shed light on the effect of dispersal limitation.

Ewens sampling formula was proposed to describe the probability distribution of alleles in genes in the context of molecular neutral evolution^56^,^58^,^59^,^60^. Hubbell^2^ applied it to compute the likelihood of a given ecological community to satisfy the prediction of the neutral theory:


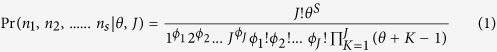


where, *J* is the total number of individuals in the community, *S* is the total number of species, *θ* is the biodiversity parameter of the sampling formula, *n*_*i*_is the abundance of species *i*, *φ*_*a*_ is the number of species with abundance *a*. Hubbell^2^ had realized the important role of dispersal limitation but the sampling formula he originally used, *i.e*., Ewens formula, did not consider the effect of dispersal limitation (*i.e*., immigration rate *m* = 1).

Etienne^57^ proposed a new sampling formula with dispersal limitation (*m* < 1),


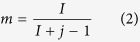


The resulting Etienne^57^ sampling formula is


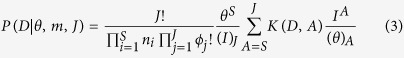


where *K*(*D*, *A*) is defined as


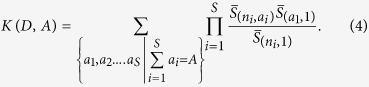


Etienne^57^ compared the two sampling formulae using Barro Colorado (BCI) tree dataset^61^ and Caño Maraca (CM) fish dataset^62^, and the results showed that Etienne sampling formula led to a significantly better fit to the datasets than Ewens sampling formula did. Here we compare the two formulae with HMP datasets to detect the potential effect of dispersal limitation. The following log-likelihood ratio test is used to compare the results from both the formulae:


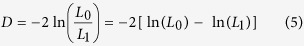
where, *L*_*0*_ is the *null* model and *L*_*1*_ is the alternative model, *D* is the deviation that is twice the difference between the log-likelihoods of the two formulae. The *p*-value computed follows a X^2^-distribution with the degree of freedom being one.

The method we use to test the community neutrality is Etienne’s^63^ “*Exact neutrality test*.” The exact neutrality test is based on the *sequential construction approach,* and an obvious advantage of this method is that no *alternative* model is needed in the approach. The test is conducted as follows: Firstly, *maximum likelihood estimation* (MLE) is used to estimate the model parameters from the observed data. Secondly, the set of model parameters [*θ*, I] estimated with MLE and parameter (*J*, the total number of individuals in the community) can be used to generate any number of artificial datasets (*D*). In our case of HMP data, 100 artificial datasets for each community sample are generated. Thirdly, we use Etienne’s formula to calculate the likelihood of artificial datasets, and compare the average likelihood of artificial data with that of the observed data.

The sampling formula [(1) or (3)] gives the probability (*P*_*S*_) of any dataset in *D* (|*D|* = 100 in our case) satisfying the neutral model. The sampling formula also gives the probability *P*_*0*_ of real observed dataset satisfying the neutral model. If the probability computed from the real dataset is significantly smaller than the average computed from the artificial datasets, it is unlikely that the observed community pattern was structured by the neutral process. If the probability from observed community is close to the values computed from the artificial datasets, then the observed species abundance distribution (SAD) is consistent with neutrality, and we conclude that the neutrality of the community under testing cannot be rejected.

## Results and Discussion

In the following, we report our results in two parts, corresponding to (*i*) the comparison of Ewens and Etienne sampling formulae, which determines which formula will be used to test the community neutrality in the next step; (*ii*) the comparison of the log-likelihoods of theoretical and observed communities to determine whether or not an observed community is neutral.

### Comparison of Ewens and Etienne sampling formulae

For each human microbial community sample, we compute the fundamental biodiversity parameter (*θ*), the immigration probability (*m*), and the likelihood of seeing the dataset given these parameter values, with Ewens and Etienne sampling formulae respectively. For each community sample, the 16s rRNA sequence data of both V1-V3 and V3-V5 variable regions were separately utilized to test the neutral theory respectively. Tables 1 and 2 are the summary results of the comparisons between Ewens and Etienne sampling formulae for region V1-V3 and V3-V5, respectively. Detailed comparative results of the two formulae are provided as supplementary Tables S1 and S2, in which parameters and statistics such as the biodiversity parameter (*θ*), the immigration probability (*m*), log-likelihood, and likelihood ratio are provided to demonstrate the suitability of the both formulae.

In Tables 1 and 1, the first column lists the five sampling location, and the second column lists the 18 sites that are distributed over the five sampling locations. Column 3 is the total number of communities for each site. Column 4 is the number of communities for which both Ewens and Etienne formulae made *no significant difference* (NSD). The results from Tables 1 and 1 show that in both V1-V3 and V3-V5 regions, there are little differences between Ewens and Etienne sampling formulae in terms of the log-likelihood (*p* > 0.05). The percentages of NSD communities in both Tables 1 and 1 are close to 100%, *i.e*., both formulae made no differences in almost all human microbial communities we tested.

### Etienne’s neutrality exact test

One hundred (100) artificial communities were generated to simulate the theoretical neutral community corresponding to each observed human microbial community. As mentioned previously, we have 2855 communities represented by V1-V3 data, and 4582 communities represented by V3-V5 data. The parameters (*m*, *θ*, *J*, *S*) of the neutral theory models for those human microbial community that have passed the *likelihood ratio test* are listed in Tables 3 and 3, for V1-V3 and V3-V5, respectively. It is noted that Etienne formula^63^ was utilized for computing the results in both Tables 3 and 3. In fact, Ewens formula could be used either since both the formulae made no difference.

Table 3 lists the parameters of 26 human microbial communities, based on V1-V3 region data, which have passed the neutrality exact test, and the results of the other 2828 communities that failed to pass the neutrality exact tests, were omitted in Table 3 (but provided in the Supplementary Table S3). It also shows that 99.1% human microbial communities based on the 16s rRNA sequence data of V3-V5 region do not satisfy the neutral community model.

Similarly, Table 4 contains the parameters of 23 human microbial communities, based on V3-V5 region data, which have passed the neutrality exact test, and the results of the other 4557 communities that failed to pass the neutrality exact tests, were omitted in Table 4 (but provided in the Supplementary Table S4). It also shows that 99.5% human microbial communities based on the 16s rRNA sequence data of V3-V5 region do not satisfy the neutral community model.

Note that among the 26 communities that passed the neutrality exact tests based on V1-V3, 21 are urological microbiome. In other words, the majority (80%) of the neutral human microbial communities are urological microbiome. Among the 23 communities that passed the neutrality exact test based on V3-V5, 14 are skin microbiome. In other words, the majority (60%) of the neutral human microbial communities are skin microbiome.

**Figure 1:**
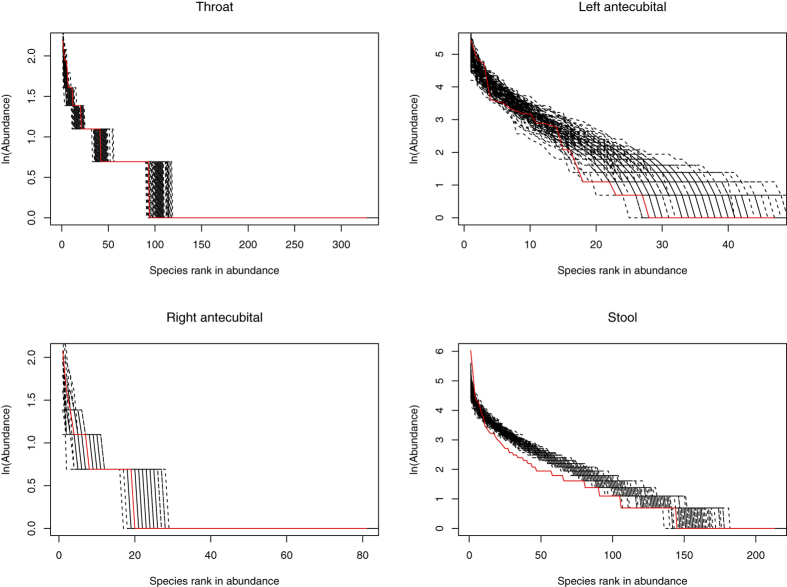
The rank abundance curves of the four samples, out of the 49 samples that were successfully fitted to Etienne’s neutral model. The four samples represent the four different sites from four different individual subjects: throat (Subject ID: 700110166), left antecubital fossa (Subject ID: 700102129), right antecubital fossa (Subject ID: 700106174), and stool (Subject ID: 700106979). In the figure, the solid red lines are observed data and the black dash lines are artificial (simulated) datasets. The *X*-axis is the species rank order in abundance and Y-axis is the abundance of each species in natural logarithm.

In summary, our results show that less than 1% (49 out of 7437 community samples) satisfied the neutral theory model according to the tests with Ewens^56^ and Etienne^57^ models. We therefore conclude that the human microbiome is not neutral in general. Figure 1 shows the graphs of four samples that successfully fit to the neutral theory model of Etienne’s^63^ neutrality exact test.

## Discussion

Bell^64^ proposed a method termed distance-decay dispersal for testing the *dispersal limitation*, which analyzes the relationship between the distance and community dissimilarity. If there is dispersal limitation effect within the community, then community dissimilarity should increase with the increase of distance, and the relationship between distance and dissimilarity should be linear^64^,^65^. Otherwise, the relationship should be non-linear. By simulation studies with two simple theoretical community models, Lotka-Volterra stochastic dynamics model and presence-absence model, Fisher & Mehta^66^ discovered that there is a phase transition in diverse ecological communities between a selection-driven regime (the niche phase) and a drift-dominated regime (the neutral phase), similar to the phase transitions occurring in H_2_O (*i.e.,* solid ice, liquid water, and gas steam). Their simulation study suggests that the niche phase is more likely in communities with large populations sizes and relative constant environments, and the neutral phase with small population sizes and fluctuating environments. The authors admitted that some simplifications, which may be unrealistic for natural ecological communities, were introduced in their study: one example is that the community is well mixed with purely competitive interactions. In reality, most natural communities are patchy, hierarchical, of complex spatial structures, and the effects of dispersal mutation, and mutualism can hardly be ignored, especially in microbial communities. Indeed, if the prediction from their simulation is taken literally, the tropical forests, which should have relatively stable environment, should be less likely to be neutral. However, it is well known that Hubbell^2^ neutral theory was strongly inspired by his studies of tropical forests. Nevertheless, we concur with Fisher & Mehta^66^ that the presence of a niche–neutral phase transition is likely robust to the additional model modifications with more realistic assumptions. We also fully agree with them that more realistic modifications are needed to develop more quantitative models for natural communities.

Inspired by Fisher & Mehta^66^ study, we propose the following hypothesis to explain the failure of neutral theory model in describing the human microbial communities. The famous microbial biogeographical doctrine first proposed by Baas-Becking^18^, “*Everything is everywhere, but the environment selects*” offers us another piece of key supporting evidence, besides Fisher & Mehta’s^66^ finding. We conjecture that the balance between dispersal and selection may tip the shift (phase transition) between the neutral and niche regimes. While dispersal favors neutral processes, selection obviously weighs against the effect of neutral processes and in favor of the niche phase.

Phylogenetic diversity has increasingly become an important tool to quantify community diversity thanks to the rapid accumulations of the metagenomic sequencing data^67^,^68^. O’Dwyer *et al*.^33^,^34^ introduced a framework that uses the phylogenetic diversity for testing the neutral theory. They compared patterns of phylogenetic diversity across multiple bacterial community samples from different habitats with the evolutionary trees generated using theoretical models of biodiversity. They obtained two major findings: the first finding is that on coarse scales the backbone of the empirical trees is simple, robust and consistent across habitats, although they are idiosyncratic on finer scales. While their first finding still supports the primary principle of the neutral theory—that selective differences are irrelevant for predicting large-scale patterns, and therefore the neutral theory is likely to predict the biodiversity patterns over coarse scales, their second finding reject the neutral theory model *per se*. They found that the existing neutral models could not explain observed patterns in microbial phylogenetic diversity, and instead, another family of Λ-coalescents models offers a qualitatively better description of both the scaling and topology of empirical trees. Their overall conclusion is that while the principle of the neutral theory is useful as a backbone for characterizing the microbial phylogenetic diversity, new generation of neutral models such as the family of Λ-coalescents models are needed to implement the utilization of the backbone for revealing the ecological and evolutionary mechanisms of microbial diversity. While we fully agree with O’Dwyer *et al*.^33^,^34^ that testing of the neutral theory with phylogenetic diversity makes great sense, especially with new type of neutral models, we suggest that different metrics for diversity may also make difference. Two biodiversity metrics should be particularly worthy of pursuing in future testing of the neutral theory. One is the UniFrac by Lozupone & Knight^67^, and another is the Hill numbers. The latter has increasingly been recognized as the most appropriate alpha-diversity metrics, and its multiplicative partition for beta-diversity is found to have excellent statistical and ecological properties advantageous over other diversity indexes^68^,^69^,^70^.

Metagenomics technology opens unprecedented opportunities to test ecological theories such as the neutral theory of biodiversity by producing gigantic amount of molecular sequencing data often from many community samples. This nevertheless also presents significant computational challenges. Some of the challenges can be dealt with standard bioinformatics software tools such as QIIME and Mothur^71^, but a far more significant challenging is to maximally take advantage of the big data of metagenomic sequences. One such challenge in the case of neutral theory modeling is the extreme computational complexity involved in multisite neutrality testing, which is computationally intractable when the number of sites (local communities) are more than a few^36^. A recent major methodological advance by Harris *et al*.^36^ offers a timely approach to the problem. The approach approximates a large class of neutral models with the hierarchical Dirichlet process by developing a highly efficient Bayesian fitting strategy for the multisite neutrality-testing problem. Besides being able to handle large datasets in a reasonable amount of time for multi-site neutrality test, an additional benefit is to generate full posterior distribution over the parameters, rather than obtaining just a maximum likelihood prediction. The approach also reconstructs the metacommunity distribution, which makes it possible to divide the problem of neutrality test into two parts. First, from the full neutral models with fitted parameters, one can generate samples and compare their likelihood with that of the observed samples to test for neutrality. Second, one can generate samples from the observed metacommunity and test for the neutrality of local community alone. Therefore, it is possible to test for neutrality at both local and metacommunity level with Harris *et al*.^36^ hierarchical Bayesian modeling approach. From a broader perspective, it is suggested that to use hierarchical Dirichlet process as an ecological null model, possibly extended to niche-neutral hybrid model and playing a more significant role in the community ecology^36^.

## Additional Information

**How to cite this article**: Li, L. and Ma, Z. (S.) Testing the Neutral Theory of Biodiversity with Human Microbiome Datasets. *Sci. Rep.* **6**, 31448; doi: 10.1038/srep31448 (2016).

## Supplementary Material

Supplementary Information

## Figures and Tables

**Table 1:** The comparison of Ewens and Etienne sampling formulae with V1-V3 region data.

Location	Site	Num. of Communities	Num. of NSD Communities	% of NSD Communities
Airways	Anterior nares	158	156	98.7
Gut	Stool	186	185	99.4
Oral	Attached Keratinized gingiva	180	179	99.4
	Buccal mucosa	181	180	99.4
	Hard palate	176	173	98.2
	Palatine Tonsils	184	182	98.9
	Saliva	159	158	99.4
	Subgingival plaque	183	183	100
	Supragingival plaque	188	187	99.5
	Throat	170	170	100
	Tongue dorsum	190	188	98.9
**Average**		179	177.8	99.3
Skin	Left Antecubital fossa	138	134	97.1
	Left Retroauricular crease	183	172	93.9
	Right Antecubital fossa	134	133	99.2
	Right Retroauricular crease	185	178	96.2
**Average**		160	154.3	96.6
Urogenital	Mid vagina	88	88	100
	Posterior fornix	86	86	100
	Vaginal introitus	86	86	100
**Average**		86.7	86.7	100

**Table 2:** The comparison of Ewens and Etienne sampling formulae with V3-V5 region data.

Location	Site	Num. of Communities	Num. of NSD Communities	% of NSD Communities
Airways	Anterior nares	259	256	98.8
Gut	Stool	318	318	100
Oral	Attached Keratinized gingiva	312	311	99.7
	Buccal mucosa	309	291	94.2
	Hard palate	301	288	95.7
	Palatine Tonsils	311	300	96.5
	Saliva	289	274	94.8
	Subgingival plaque	307	305	99.3
	Supragingival plaque	313	309	98.7
	Throat	304	291	95.7
	Tongue dorsum	315	311	98.7
**Average**		306.8	297.8	97.03
Skin	Left Antecubital fossa	147	147	100
	Left Retroauricular crease	271	270	99.6
	Right Antecubital fossa	154	152	98.7
	Right Retroauricular crease	283	280	98.9
**Average**		213.8	212.2	99.3
Urogenital	Mid vagina	133	133	100
	Posterior fornix	133	132	99.2
	Vaginal introitus	123	123	100
**Average**		129.7	129.3	99.7

**Table 3:** The 26 human microbial communities that passed the neutrality exact tests with Etienne sampling formula (V1-V3 region)*.

Site	ID	*J*	*S*	*θ*	*m*	Log(L_0_)	Log(L_1_)	*q*-value	*p*-value
Throat	700110166	506	327	399.09	0.999978	−16.98	−15.5	2.962	0.0852
Left Antecubital fossa	700102129	808	47	10.73	0.999980	−57.89	−56.6	2.594	0.1072
Left Antecubital fossa	700113117	1462	80	18.05	0.999812	−79.40	−79.9	1.078	0.2990
Right Antecubital fossa	700106174	115	81	120.54	0.999998	−9.24	−8.2	2.088	0.1485
Right Antecubital fossa	700105936	139	16	4.47	0.995735	−19.55	−20.3	1.416	0.2341
Mid vagina	700015239	12512	343	65.04	0.979742	−249.36	−251.1	3.547	0.0597
Mid vagina	700016499	25053	498	88.27	0.999139	−359.68	−358.0	3.359	0.0668
Mid vagina	700110832	4375	164	33.58	0.999910	−144.54	−144.1	0.960	0.3272
Mid vagina	700023176	5815	168	32.25	0.999892	−163.09	−163.9	1.598	0.2062
Mid vagina	700033039	4670	160	31.90	0.999952	−148.08	−148.3	0.526	0.4683
Posterior fornix	700109404	4090	174	36.77	0.997910	−140.96	−139.9	2.089	0.1484
Posterior fornix	700110833	3537	145	30.25	0.999471	−127.48	−129.3	3.672	0.0553
Posterior fornix	700024357	8120	181	32.64	0.999853	−194.34	−192.9	2.875	0.0900
Posterior fornix	700024242	7460	179	32.85	0.998841	−186.94	−185.1	3.603	0.0577
Posterior fornix	700106808	10460	288	54.50	0.999765	−228.76	−227.0	3.474	0.0623
Posterior fornix	700023398	3980	153	31.39	0.999889	−135.71	−136.9	2.352	0.1251
Posterior fornix	700015577	8946	243	46.00	0.999900	−209.83	−208.0	3.618	0.0572
Vaginal introitus	700015455	9337	294	57.53	0.999909	−213.75	−215.1	2.673	0.1021
Vaginal introitus	700023640	7943	259	51.11	0.999965	−198.53	−197.5	2.011	0.1562
Vaginal introitus	700023453	8425	242	46.41	0.999901	−202.07	−202.6	0.973	0.3240
Vaginal introitus	700114121	6847	223	44.18	0.997384	−181.00	−182.7	3.437	0.0637
Vaginal introitus	700105207	7311	218	42.08	0.999904	−188.58	−188.3	0.475	0.4906
Vaginal introitus	700109402	4418	224	49.60	0.999936	−146.13	−145.6	1.095	0.2954
Vaginal introitus	700098286	2679	223	58.57	0.902479	−109.72	−108.7	2.020	0.1553
Vaginal introitus	700097310	2277	123	27.66	0.999520	−103.66	−102.9	1.446	0.2292
Vaginal introitus	700114273	1934	195	53.86	0.999516	−89.83	−89.4	0.899	0.3431

^*^J: the total number of reads in the sample, *S*: the number of species in the sample, *θ*: fundamental biodiversity, *m*: immigration probability, Log(L_0_) is the log-likelihood of the observed sample, Log(L_1_) is the log-likelihood predicted by the neutral model, and *q-*value and *p*-value are the values of the likelihood ratios.

**Table 4:** The 23 human microbial communities that passed the neutrality exact tests with Etienne sampling formula (V3-V5 region)*.

Site	ID	*J*	*S*	*θ*	*m*	Log(L_0_)	Log(L_1_)	*q*-value	*p*-value
Anterior nares	700105415	130	93	144.71	0.999995	−8.40	−9.67	2.5417	0.1109
Stool	700106979	413	292	2029.70	0.591395	−12.16	−10.51	3.3049	0.0691
Saliva	700106858	227	144	168.36	0.999857	−12.41	−13.24	1.6687	0.1964
Saliva	700106000	161	101	183.19	0.703965	−10.93	−11.94	2.0187	0.1554
Left Antecubital fossa	700021896	377	82	32.01	0.999912	−34.90	−36.18	2.5604	0.1096
Left Antecubital fossa	700024590	173	27	8.73	0.999985	−24.35	−24.39	0.0667	0.7961
Left Antecubital fossa	700021810	364	81	31.99	0.999979	−33.97	−35.23	2.5302	0.1117
Left Retroauricular crease	700037027	110	14	4.04	0.999998	−18.16	−17.60	1.1143	0.2912
Left Retroauricular crease	700024129	199	51	21.84	0.999893	−24.01	−23.59	0.8559	0.3549
Left Retroauricular crease	700024647	174	31	10.72	0.999990	−24.40	−23.35	2.0904	0.1482
Right Antecubital fossa	700110106	165	119	190.08	0.999997	−9.16	−10.61	2.9033	0.0884
Right Antecubital fossa	700105617	128	21	14.37	0.166726	−20.11	−18.31	3.6134	0.0573
Right Antecubital fossa	700037030	114	38	19.55	0.999967	−16.44	−16.00	0.8825	0.3475
Right Antecubital fossa	700035745	329	37	10.44	0.985781	−35.34	−34.06	2.5613	0.1095
Right Antecubital fossa	700015672	331	106	53.53	0.999989	−28.24	−30.15	3.8298	0.0503
Right Antecubital fossa	700023394	382	62	20.76	0.999987	−37.39	−36.55	1.6819	0.1947
Right Antecubital fossa	700023241	922	95	26.36	0.999952	−62.30	−62.60	0.6052	0.4366
Right Retroauricular crease	700023171	641	111	38.46	0.999779	−47.49	−47.16	0.6634	0.4154
Mid vagina	700099742	4365	183	38.45	0.999805	−144.58	−146.30	3.4341	0.0639
Posterior fornix	700110242	3419	155	33.31	0.999917	−127.00	−125.46	3.0837	0.0791
Posterior fornix	700105684	3803	156	32.65	0.999534	−133.92	−132.29	3.2624	0.0709
Posterior fornix	700097502	2983	163	36.92	0.999941	−118.04	−118.87	1.6699	0.1963
Posterior fornix	700099080	5079	175	34.93	0.999892	−155.68	−156.69	2.0075	0.1565

^*^J: the total number of reads in the sample, *S*: the number of species in the sample, *θ*: fundamental biodiversity, *m*: immigration probability, Log(L_0_) is the log-likelihood of the observed sample, Log(L_1_) is the log-likelihood predicted by the neutral model, and *q-*value and *p*-value are the values of the likelihood ratios.
